# Increased occurrence of spinal fractures related to ankylosing spondylitis: a prospective 22-year cohort study in 17,764 patients from a national registry in Sweden

**DOI:** 10.1186/1754-9493-7-2

**Published:** 2013-01-07

**Authors:** Yohan Robinson, Bengt Sandén, Claes Olerud

**Affiliations:** 1 Department of Orthopaedics, Uppsala University Hospital, Institute for Surgical Sciences, Uppsala, 75185, Sweden

**Keywords:** Ankylosing spondylitis, Spinal fractures, Epidemiology, Injury prevention

## Abstract

**Background:**

Ankylosing spondylitis (AS) is a rheumatoid disease leading to progressive ossification of the spinal column. Patients suffering from AS are highly susceptible to unstable vertebral fractures and often require surgical stabilisation due to long lever arms. Medical treatment of these patients improved during the last decades, but until now it is unknown whether the annual number of spinal fractures changed during the last years. Since the annual count of fracture is an effective measure for efficacy of injury prevention and patient safety in AS patients, the current recommendations of activity have to be revised accordingly.

**Methods:**

Data for all patients with AS treated as inpatients between 01/01/1987 and 31/12/2008 were extracted from the Swedish National Hospital Discharge Registry (SNHDR). The data in the registry are collected prospectively, recording all inpatient admissions throughout Sweden. The SNHDR uses the codes for diagnoses at discharge according to the Swedish versions of the International Classification of Diseases (ICD-9 and ICD-10).

**Results:**

During the years from 1987 to 2008 17,764 patients with AS were treated as inpatients; of these 724 patients were treated due to spinal fractures. The annual number of cervical, thoracic and lumbar fractures in the registry increased until 2008 (r = 0.94).

**Conclusions:**

Despite the improved treatment of AS the annual number of vertebral fractures requiring inpatient care increased during the last two decades. Possible explanations are population growth, greater awareness of fractures, improved diagnostics, improved emergency care reducing fatalities, and a higher activity level of patients receiving modern medical therapy. Obviously the improvement of medical treatment did not reduce the susceptibility of these patients to unstable fractures. Thus the restrictive injury prevention recommendations for patients with AS cannot be defused, but must be critically revised to improve patient safety.

## Background

Ankylosing spondylitis (AS) is a rheumatoid disease leading to increased stiffness and eventually to a spontaneous fusion of all spinal segments from skull base to os ilium. Due to reduced biomechanical flexibility the spine is experiencing long lever arms even when under minor trauma [1], rendering it highly susceptible to unstable vertebral fractures. The prevalent osteoporosis in AS adds to the risk of fractures [2]. A recent retrospective cohort study in more than 230,000 patients found a 3.3 times greater risk of vertebral fractures in patients with AS than in healthy patients [3]. These fractures are associated with an increased mortality up to two years after fracture [4,5].

Biomechanically surgical treatment of spinal fractures related to AS requires neutralisation of the lever arms – commonly performed by multilevel internal fixation [6]. If not adequately stabilised, these patients may experience pseudarthrosis, implant failure, and secondary dislocation [7]. Medical treatment of AS improved dramatically during the last decades. Biological treatment with anti tumour necrosis-factor and optimised treatment with disease modifying anti-rheumatic drugs lead to a less symptomatic disease, and early physiotherapy may delay onset of stiffness [8,9].

A large patient registry-based analysis from the UK found a decreased risk for vertebral fractures, if patients with AS were taking non-steroidal antirheumatic drugs (NSAR) [3]. Despite this finding it is still unclear whether the improved treatment leads to a reduced annual number of spinal fractures. Control of the fracture-risk with medication and injury prevention is an important public health issue, and efficacy of fracture prevention should be investigated regularly by epidemiological means. National registries as the Swedish National Hospital Discharge Registry are excellent tools to investigate these epidemiological hypotheses.

## Methods

Sweden has a national public healthcare system, based on independent county councils, mainly financed by local taxes.

The Swedish National Board of Health and Welfare registers data on hospital discharges in the Swedish National Hospital Discharge Registry (SNHDR). Each record contains information about demographics, diagnoses, operations, and administrative data about the healthcare provider and the admission [10,11]. Since 1987 more than 99% of all discharges are registered, and each year about 800,000 discharges are recorded (Table 1) [10,11]. The validity of the data in the registry has previously shown to be adequate [10,12]. 

**Table 1:** Swedish national population data from the central bureau of Statistics Sweden, total patient count, and annual number of patients with ankylosing spondylitis and vertebral fractures in the SNHDR

**Year**	**1987**	**1988**	**1989**	**1990**	**1991**	**1992**	**1993**	**1994**	**1995**	**1996**	**1997**	**1998**	**1999**	**2000**	**2001**	**2002**	**2003**	**2004**	**2005**	**2006**	**2007**	**2008**
**population of Sweden**	8414083	8458888	8527036	8590630	8644119	8692013	8745109	8816381	8837496	8844499	8847625	8854322	8861426	8882792	8909128	8940788	8975670	9011392	9047752	9113257	9182927	9256347
**- inpatients**	882217	890888	877573	882120	911614	899085	899100	885271	853826	836382	819397	813845	798583	787545	775556	771837	773543	776636	785526	798290	797357	803128
**- all musculoskeletal diagnoses**	64431	62847	62047	61594	66150	69730	68993	67297	63365	63054	63225	63451	60335	59913	60152	60442	62295	64202	65831	67730	67496	69378
**- all ankylosing spondylitis (AS)**	729	662	746	666	690	787	818	823	796	803	737	744	661	624	650	759	826	883	982	1037	1115	1226
**- all fractures with AS**	6	9	6	9	4	21	22	29	30	37	40	29	50	52	77	59	67	89	90	93	78	139
**- cervical spine fractures and AS**	4	4	3	5	3	10	9	13	11	14	22	11	20	20	32	26	31	31	37	27	27	40
**- thoracic spine fractures and AS**	0	3	3	1	0	2	2	6	7	9	3	8	10	9	12	12	6	15	13	25	15	35
**- lumbar spine fractures and AS**	1	2	0	1	0	7	10	4	4	7	7	3	9	8	9	4	6	10	8	6	9	13

All patients with a primary discharge diagnosis of cervical, thoracic or lumbar vertebral fractures, spine dislocations, or disc ruptures were identified in the SNHDR (International Classification of Diseases (ICD)-9: 805.0, 805.1, 805.2, 805.3, 805.4, 805.5; ICD-10: S12.0, S12.1, S12.2, S22.0, S22.1, S23.0, S23.1, S32.0, S32.1, S33.0, S33.1) [13,14]. Furthermore all patients with the diagnosis of ankylosing spondylitis were identified (ICD-9: 720.0, ICD-10: M45). The ICD-9 was used until 31/12/1996, since 01/01/1997 the ICD-10 was used to code the discharge diagnosis.

Annual patient counts of interest were extracted by the Centre of Epidemiology of the Swedish National Board of Health and Welfare (http://www.socialstyrelsen.se), and population data was obtained by reports from the central bureau of Statistics Sweden (http://www.scb.se). Data collection and statistical evaluation was performed using the IBM SPSS Statistics Software (Version 19.0.0).

The study was approved by the Regional Research Ethics Board in Uppsala (dnr 2010/297).

## Results

Between 01/01/1987 and 31/12/2008 a total number of 17764 patients with AS were registered as inpatients. The annual hospitalisation number until 2000 showed a relativly stable interval between 624 and 823 patients, but from 2001 onward a significant linear increase from 650 in 2001 to 1226 in 2008 (r = 0.99) was seen (Table 1). The population in Sweden grew between 1987 and 2008 at a rate of 0.46% every year.

**Figure 1:**
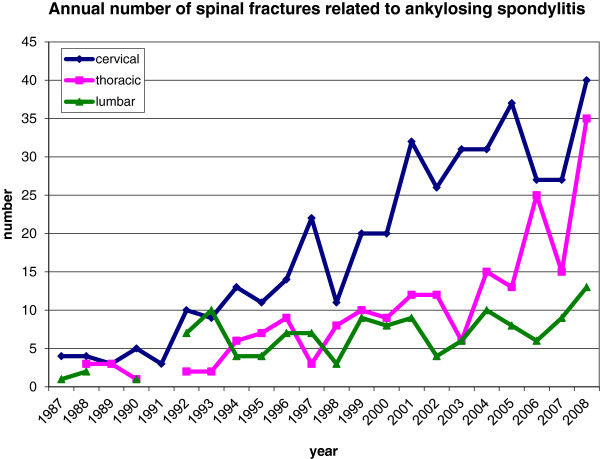
Annual number of spinal fractures related to AS according to the region of the spinal column.

About 4.1% (n = 724) of patients with AS these had fractures of the spine. There was a significant positive linear trend in the annual count of AS-patients with spinal fractures (r = 0.94). This was more distinct for cervical (r = 0.82) and thoracic fracures (r = 0.85) than for lumbar fractures (r = 0.56) (Figure 1). The proportion of spinal fractures in admitted AS patients increased starting from 0.82% in 1987 up to 11.3% in 2008.

## Discussion

During the last two decades a steady increase in the annual number of patients with spinal fractures related to AS was found in Sweden. Although it has been suggested that through better medical treatment of AS the risk of suffering an unstable fracture should be reduced, this has not been found in this analysis of this registry data. The data from the SNHDR does not allow similar conclusions as a recently published study in 758 patients with AS by Vosse et al. [3] presenting reduced fracture risk if the patients were receiving medical therapy (OR 0.65). Swedish healthcare resource utilisation in patients with AS did not differ from other developed countries with 6–7 physician visits annually, thus improved medical therapy according to international guidelines should be expected during the last decades [15]. Thus either the effect of medical therapy has not reached epidemiological significance, yet, or other underlying factors have to be put into consideration.

The first and possibly most obvious explanation would be an observational error, meaning a systematic bias during data collection. A recently published review on the SNHDR found high validity especially for surgical diagnoses [10]. Validation of the SNHDR using national quality registries demonstrated that impressive 94% of all knee arthroplasty cases, 93% of all hip arthroplasty cases, and 95% of all hip fracture cases were correctly identified within the SNHDR. Similar numbers can be assumed for other orthopaedic diagnoses as vertebral fractures and AS. Furthermore in Sweden full reimbursement for treatment requires registration of codes for diagnosis and treatment in the National Hospital Discharge Registry. Therefore it can be assumed that coding errors are minimal.

A second possible reason for increased numbers of spinal fractures related to AS is an improved survival of patients suffering from unstable vertebral fractures. The metaanalysis of Westerveld et al. [4] found an overall mortality of 17.7% within the first three months after a vertebral fracture in AS, being 6.4% in the operatively treated and 11.3% in the non-operatively treated subgroup (n.s.). A recently published survival study by Schoenfeld et al. [5] found an increased mortality in patients with ankylosing spondylitis compared to controls even at time points up to 2 years after fracture. Optimised acute treatment during the last decades may have led to improved survival directly after injury, leading to more hospital admissions being registered in the National Hospital Discharge Registry. Unfortunately there is no data in the SNHDR on the mortality during the immediate post-injury phase.

A further explanation for the increase of spinal fractures in AS is an increasing level of activity with a reduced safety profile and greater risk for injuries. Multiple medical treatment strategies as well as physiotherapy interventions have been found to improve function and reduce stiffness in AS [8,9]. It can be assumed that a new generation of patients with AS appeared during the last decades, being early under medical treatment and receiving optimised physiotherapy. These patients very likely have a quality of life similar to healthy individuals and experience ankylosis much later in life and at a much lower degree of kyphosis [16]. Unfortunately once the biomechanical flexibility of the spine is declining, these still very active patients are prone to injuries, possibly leading to a greater number of spinal fractures [17].

Beyond that it is possible that patients with AS have nowadays a prolonged life span due to improved therapy. This would cause an increasing population of patients with AS due to reduced mortality. This hypothesis is supported by the finding that the number of registered patients with AS increased during the observed years (r = 0.67) (Table 1).

Finally, improved diagnostics identify earlier and more accurately AS. In the National Hospital Discharge Registry a relatively unchanged annual number of spinal fractures related to ankylosing spondylitis was seen until 2000. From 2001 onward a significant linear increase was found, suggesting either a greater spread of the disease, or - rather more likely – improved diagnosis of AS. The greater implementation of the New York-criteria for diagnosis of AS allowed a more standardised and homogenous identification of the disease [18]. Possibly a certain number of patients with AS and vertebral fractures did not enter the study because the disease was not identified, yet.

The observed increase in total numbers of vertebral fractures in all regions of the spine is accompanied by a relative decline in cervical and lumbar fractures in favour of thoracic fractures (Table 1, Figure 1). In the last years there has been a positive trend towards the use of computed tomography instead of conventional radiographs especially in patients with AS [7]. A decreased number of missed fractures could explain the relative increase in thoracic fractures, which can be hard to visualise on plain radiographs. Other possible explanations as a change in trauma patterns, and reduced ankylosis of cervical and lumbar spine are only hypothetical and lack any supporting evidence.

With regard to activity recommendations there exist diverging opinions on restrictions, but most authors agree that following general safety precautions are valid [17,19,20]: 1. The excessive use of alcohol should be avoided. 2. Contact sports (i.e. rugby, martial arts, ice hockey) should be avoided. 3. High impact sports (i.e. tennis, soccer) are not recommended during an acute inflammatory phase and in protracted stages of the disease. 4. Seat belts and car seat headrests should be used at all times while driving. Due to the increasing ankylosis and secondary osteoporosis restrictions for physical activity weigh stronger the older the patient is, and the longer he is suffering from AS.

## Conclusions

Regardless of the underlying causes an obvious increase in the number of spinal fractures in patients with AS could be observed. Even though the medical therapy changed the course of the disease dramatically, it seems that the improved quality of life and function in these patients does not correlate with a reduced fracture risk. Thus medical practitioners are urged to inform all patients with AS to keep a high safety profile when engaging in sports or other activities that could lead to injuries due to the increased fracture risk.

## Competing interests

The authors declare that they have no competing interests.

## Authors’ contributions

YR designed the study, performed the statistical analysis, and wrote the manuscript. BS and CO revised the manuscript critically. All authors read and approved the final manuscript.
